# Osteoconductive and electroactive carbon nanofibers/hydroxyapatite nanocomposite tailored for bone tissue engineering: in vitro and in vivo studies

**DOI:** 10.1038/s41598-020-71455-3

**Published:** 2020-09-09

**Authors:** Hadi Samadian, Hamid Mobasheri, Mahmoud Azami, Reza Faridi-Majidi

**Affiliations:** 1 Nano Drug Delivery Research Center, Health Technology Institute, Kermanshah University of Medical Sciences, Kermanshah, Iran; 2 Laboratory of Membrane Biophysics and Macromolecules, Institute of Biochemistry and Biophysics, University of Tehran, Tehran, Iran; 3 Institute of Biomaterials, University of Tehrand and Tehran University of Medical Sciences (IBUTUMS), Tehran, Iran; 4 Department of Tissue Engineering and Applied Cell Sciences, School of Advanced Technologies in Medicine, Tehran University of Medical Sciences, Tehran, Iran; 5 Department of Medical Nanotechnology, School of Advanced Technologies in Medicine, Tehran University of Medical Sciences, Tehran, Iran

**Keywords:** Nanomedicine, Nanoscale materials

## Abstract

In this study, we aimed to fabricate osteoconductive electrospun carbon nanofibers (CNFs) decorated with hydroxyapatite (HA) crystal to be used as the bone tissue engineering scaffold in the animal model. CNFs were derived from electrospun polyacrylonitrile (PAN) nanofibers via heat treatment and the carbonized nanofibers were mineralized by a biomimetic approach. The growth of HA crystals was confirmed using XRD, FTIR, and EDAX analysis techniques. The mineralization process turned the hydrophobic CNFs (WCA: 133.5° ± 0.6°) to hydrophilic CNFs/HA nanocomposite (WCA 15.3° ± 1°). The in vitro assessments revealed that the fabricated 24M-CNFs nanocomposite was biocompatible. The osteoconductive characteristics of CNFs/HA nanocomposite promoted in vivo bone formation in the rat’s femur defect site, significantly, observed by computed tomography (CT) scan images and histological evaluation. Moreover, the histomorphometric analysis showed the highest new bone formation (61.3 ± 4.2%) in the M-CNFs treated group, which was significantly higher than the negative control group (the defect without treatment) (< 0.05). To sum up, the results implied that the fabricated CNFs/HA nanocomposite could be considered as the promising bone healing material.

## Introduction

Advanced bioactive materials play a central role as supporting scaffolds in bone tissue engineering approaches. These materials provide efficient support for bone growth and stimulate the body's constructive biological responses to accelerate the healing process^1–3^. A proper scaffold must resemble fine structures of the bone tissue and possess its various characteristics, including mechanical strength, as well as biological, chemical, and electrical properties^4,5^. Bone is an electroactive and well organized architectural structure that spans from nano to macro-scale dimensions^6,7^. Moreover, the healing process initiates, controls, and progresses from nano to macro levels. Scaffolds with well-defined nanostructures, thus, possess promising features to support, guide and accelerate the healing process. Hence, a great deal of effort is being applied to develop and tailor appropriate scaffolds for bone tissue engineering purposes^8^.

Bone is a metabolically dynamic and electroactive tissue containing various cell types arranged in a specialized matrix. It is a natural composite mainly comprised of a network of collagen nanofibers decorated and reinforced by different calcium/phosphate-based minerals, including hydroxyapatite (HA)^9,10^. The HA bioceramic contributes to the mechanical strength of bone and is involved in many physiological and metabolic processes. HA molecules act as a reservoir for calcium (Ca) and phosphorus (P) and are responsible for osteoconduction, and osteoinduction^11^. Inspired by the structure of bone, various bioceramic based nanocomposite, with dispersed and/or continuous phase nanostructures have been constructed and proposed for bone tissue engineering. Mimicking the fibrous structure of the extracellular matrix (ECM) in bone, various nanofibers from different sources have been utilized as a scaffold to promote the bone healing process^12,13^.

Electro-conductive nanofibers provide both nanofibrous structure and electro-activity, which synergically improve the healing process^14^. There is a growing body of evidence reporting the promising potential of electro-conductive nanomaterials in bone tissue engineering applications^15,16^. Electrospun carbon nanofibers (CNFs) have found myriad applications in electroactive tissue engineering, particularly for bone tissue engineering, due to their extraordinary properties. CNFs possess several constructive characteristics that matter in tissue engineering, including; nanometric diameter, excellent mechanical strength, comparable electrical conductivity, chemical inertness, and biocompatibility. Moreover, CNFs circumvent some drawbacks of CNTs, such as potential toxicity caused by reactive oxygen species (ROS) production^17^. Generally, electrospun CNFs are fabricated from various polymeric precursors, including; poly(acrylonitrile) (PAN), pitch, lignin, and Polyimide (PI) via heat treatment in inert gas. Among them, PAN is the most popular due to its high carbon yield (more than 50 wt.%), excellent mechanical properties, and appropriate electrical conductivity^18,19^. The application of electrospun CNFs for nerve tissue engineering through differentiation of mesenchymal stem cell to neural cells has been well documented by our group^20^. Rajzer and colleagues have also fabricated CNFs from PAN/HA nanofibers and used them as a scaffold for bone tissue engineering^21^. They reported that the fabricated CNFs were bioactive and useful for bone tissue engineering applications.

The combination of nanofibers with calcium phosphate-based bioceramics has shown to be a promising strategy for mimicking the native structure of bone. Accordingly, various nanofibers have been composited with a variety of bioceramics such as bioactive glass, alumina, Zirconia, glass ceramics, resorbable calcium phosphates, and HA^22,23^. The mentioned bioceramics further improved the biocompatibility, osteoconductivity, and osteoinductivity of the resulted scaffold^24^. Amongst them, HA has drawn considerable attention due to its resemblance to the inorganic phase of the bone. Furthermore, the synergic effect of HA crystals with electro-conductive nanofibers in the resulting CNFs-HA nanocomposites would positively affect the bone fracture healing process. The CNFs-HA nanocomposite has used as a substrate for cell growth and differentiation and also served as the bone void filler in the clinical applications^25,26^. Therefore, we envisioned developing the effective bioactive bone healing material constructed from electro-conductive CNFs and composited with biomimetic HA crystals as potential scaffolds for bone tissue engineering.

## Results and discussion

### CNFs characterization

#### Morphological observation

**Figure 1:**
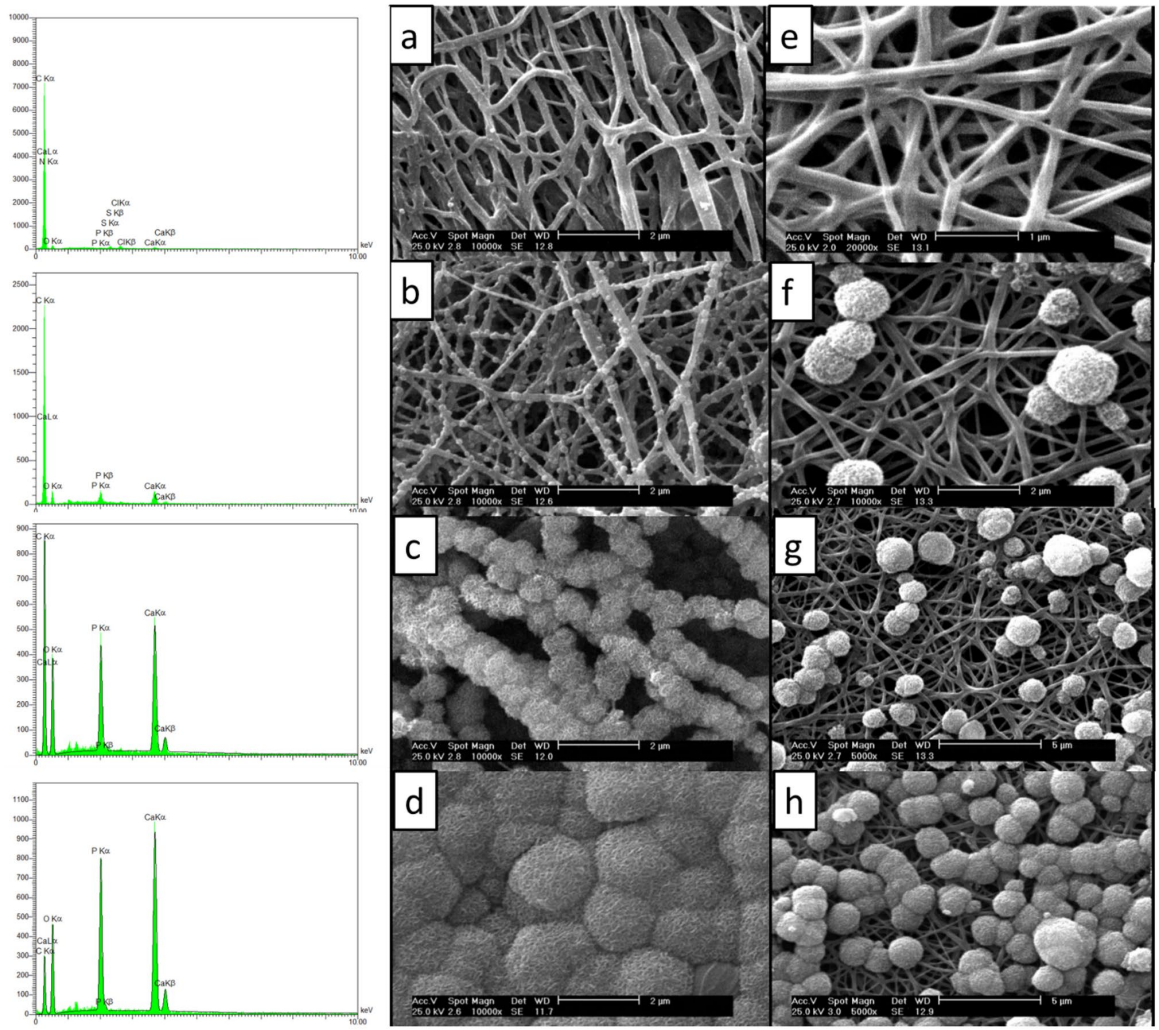
SEM micrographs of P-CNFs and T-CNFs incubated in SBF. (**a**) T-CNFs-12 h, (**b**) T-CNFs-24 h, (**c**) T-CNFs-48 h, (**d**) T-CNFs-72 h, (**e**) P-CNFs-12 h, (**f**) P-CNFs-24, (**g**) P-CNFs-48 h, and (**h**) P-CNFs-72 h.

The mineralization was conducted on pristine (P-CNFs) and treated CNFs (T-CNFs) and the formation of the mineral crystal on the surface of CNFs was visualized by SEM (Fig. 1). The surface activation was performed to induce carbonyl functional groups on the CNFs surface to serve as the mineralization site. Due to the negative charge, the induced carbonyl groups were able to interact with positively charged ions such as Ca^2+^ in the SBF solution and formed the site for mineral phase nucleation^27^.

As shown in Fig. 1, the heterogeneity of the crystal formation on the surface of CNFs was significantly different in P-CNFs and T-CNFs samples. The crystal formation on the T-CNFs was homogenous and nearly a uniform crystal layer completely covered CNFs after 48 h (Fig. 1b). On the other hand, the crystal on the P-CNFs was formed heterogeneously and caused separated granules of the mineral phase. In other words, the nucleation sites on the P-CNFs are not homogenous and crystals are formed as separated granules with different sizes. The uniform mineralization can be due to the induction of COO^-^ on the CNFs surface, which formed a homogenous nucleation sites for crystals deposition.

Moreover, the crystals are only formed on the outer layer of the P-CNFs mat, while the deeper layers of CNFs are still bare without any observable mineral phase (Fig. 1). This crystal growth pattern can be attributed to the hydrophobic nature of P-CNFs, which prohibited the movement of SBF from the surface to the depth of the nanofibrous mat. On the other hand, according to wettability measurements, the treatment converted the hydrophobic nature of CNFs to a hydrophilic one (Table 1). These observations are in agreement with the previous works conducted on mineralized carbon nanomaterials. Wu et al. fabricated CNFs and used a biomimetic approach using the SBF solution to grow HA crystals on the CNFs. They observed that the hydrophilicity of the nanofibers could control the mineralization process (growth of HA on the CNFs surface)^27^. In another study, Zhao et al.^28^ reported that the surface functionalization of CNTs affected the self-assembly of HA and induction of the negatively charged functional groups initiated the HA crystal formation. They functionalized CNTs with phosphonates and poly(aminobenzene sulfonic acid) (PABS) and observed that HA crystals nucleated and crystallized on the surface of functionalized CNTs. Moreover, they pointed out that the thickness of the HA layers directly correlated with the mineralization time.

**Table 1:**
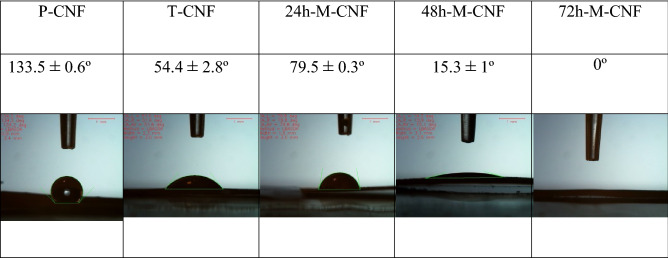
Water contact angle value of CNFs.

#### Mineral phase identification

**Figure 2:**
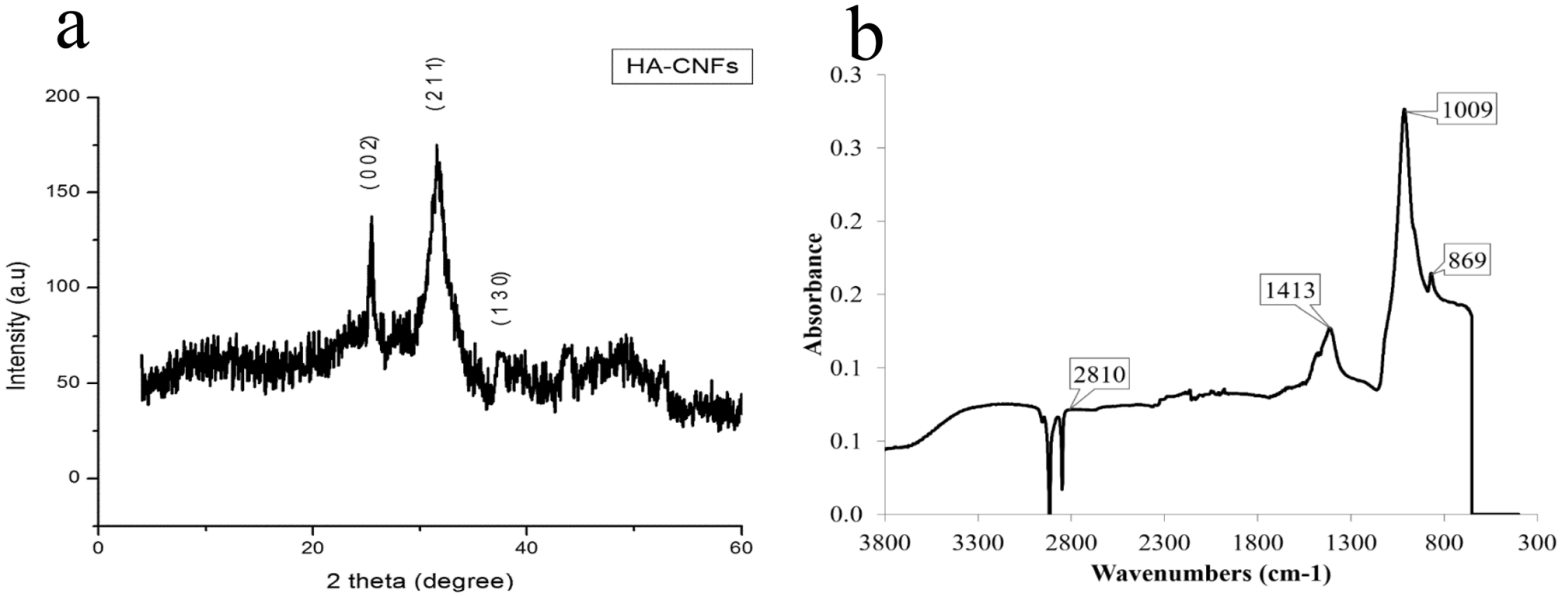
(**a**) XRD patterns and (**b**) FTIR spectra of M-CNFs nanocomposite.

The mineral phase formed on the surface of CNFs was characterized using XRD, FTIR, and EDAX analysis. The XRD pattern was recorded to identify the crystallographic nature of M-CNFs nanocomposite and the results are presented in Fig. 2a.

As shown in Fig. 2a, there are two sharp peaks around 26° and 32°, which are related to the (002) and (112) crystalline planes of HA crystals, respectively^29^. These results clearly indicated the formation of HA crystals on the CNFs. Moreover, according to the calculation based on the Sherrer equation the average crystallite size of the mineral phase was 35.2 nm, based on the half-width of the (112) reflection peak. This size is comparable with the crystal size of HA in the natural bone, 10–50 nm^30^. Moreover, the precipitated HA crystals have low crystallinity, according to the results of XRD diffractogram. Rusu et al.^31^ applied a stepwise co-precipitation method to grow HA in the Chitosan matrix using calcium, phosphate, and sodium-rich solution in the alkaline condition. They also reported a synthesized HA with a size of 15–50 nm. Furthermore, according to our previous observations^32^, the peaks related to pristine CNFs are buried beneath the peaks of mineralized CNFs.

The mineral phase grown on the CNFs was further analyzed by FTIR spectroscopy (Fig. 2b). The wide peak appeared at 3,200–3,500 cm^−1^ is related to the stretching vibration of hydroxyl groups. The peaks located at 869 and 1,413 cm^−1^ contribute to the plane bending and stretching vibration of carbonate groups (CO_3_^2−^)^33^. These results confirmed that the mineral phase grown on the CNFs is carbonate-containing HA. Previous biomimetic studies by SBF have also reported the formation of the carbonate-containing HA mineral phase^34,35^. It was shown that carbonate substituted HA was structurally and chemically similar to the natural bone^36^.

**Figure 3:**
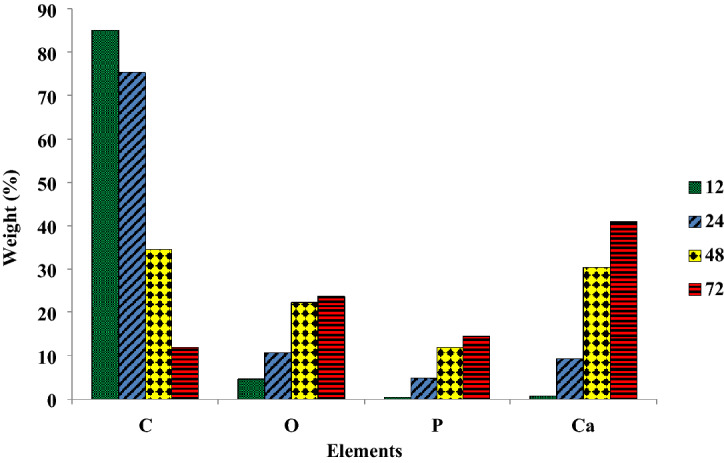
Elemental analysis of M-CNFs at different incubation time (hours) conducted by EDAX.

EDAX elemental analysis showed that the grown mineral phase was composed of O, P, and Ca, which are the main elements of HA crystal (Fig. 3). Moreover, it is apparent that increasing the incubation time enhanced the mineral phase formation on the CNFs (Fig. 5). These observations were consistent with the SEM images. The stoichiometric Ca/P ratios for 12M-CNFs, 24M-CNFs, 48M-CNFs, 72M-CNFs were 1.75, 1.92, 2.55, and 2.80, respectively.

#### Mechanical properties evaluation

**Figure 4:**
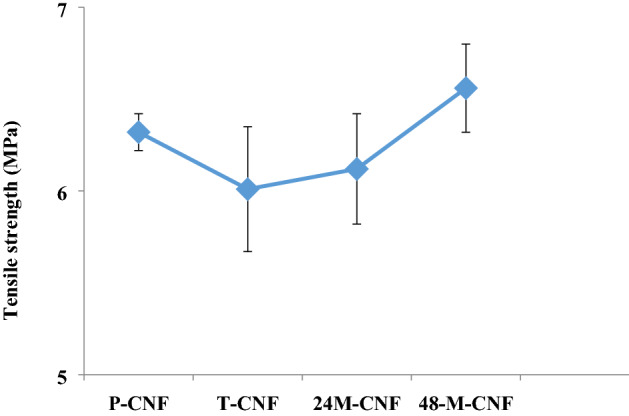
Mechanical property of CNFs, measured by the tensile strength method.

The tensile strength measurement was conducted to evaluate the mechanical properties of the prepared CNFs. The results showed that the treatment with NaOH reduced the tensile strength from 6.32 ± 0.10 to 6.01 ± 0.34 MPa. This reduction in mechanical property is related to the introduced defects in the structure of CNFs due to the NaOH treatment. As shown in Fig. 4, the mineralization enhanced the tensile strength, slightly after 24 h, but significantly after 48 h incubation. The increased mechanical property can be attributed to the reduced porosity of M-CNFs mate. It has been shown that the porosity has a reverse correlation with the mechanical properties of nanofibrous mat^37^. During the mineralization, HA crystals grow on the CNFs and partially fill the pores between nanofibers, which subsequently improve the mechanical properties. The same observation was reported by Liu et al.^38^, who mineralized electrospun poly(lactic-co-glycolic acid) (PLGA) nanofibers by concentrated SBF (10 ×). They observed that the modulus greatly increased with the mineralization process. In another study, Xie et al.^39^ incorporated HA onto the electrospun poly(ε-caprolactone) nanofibers using SBF. They also reported that the mineralization improved the mechanical properties of the nanofibers.

#### Wettability assessment

Wettability of CNFs was measured based on the water contact angle (WCA) measurement. The results indicated that P-CNFs are hydrophobic, with the WCA value of 133.5° ± 0.6°. As shown in Table 1, NaOH treatment enhanced the hydrophilicity of CNFs and reduced the WCA to 54.4° ± 2.8°, indicating that the treatment altered surface chemical structure (Table 1). The hydrophilic surface is substantial for HA crystal growth. It is interesting to find that the mineralization for 24 h increased the hydrophobicity of CNFs compared with T-CNFs. This observation can be related to the surface roughness of CNFs. As shown in Fig. 2, due to HA crystal growth after 24 h, the surface rashness increased, which subsequently increased the hydrophobicity^40^. Further HA crystal growth and full coverage of the CNFs significantly decreased the WCA. These observations indicated that the hydrophobic P-CNFs turned to the highly hydrophilic structure. The WCA values for 48 h-M-CNF and 72 h-M-CNF were 15.3° ± 1.0° and 0°, respectively. Interestingly, the water droplet was instantly absorbed into the 72 h-M-CNF rather than spreading on the porous mat.

#### Biodegradation measurement

**Figure 5:**
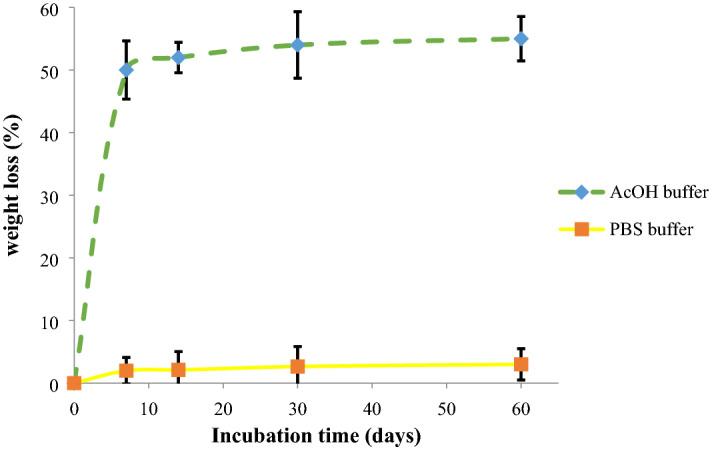
Biodegradation rate measurement in PBS and AcOH buffer during 60 days.

The degradation rate of CNFs was recorded in two different solutions to mimic two distinct physiological processes. PBS solution (pH 7.4) was used to mimic the normal physiological condition and the degradation results showed that CNFs are non-degradable in the normal condition (Fig. 5). Moreover, it is known that bone-resorbing cells, osteoclasts, are actively resorbed by bone tissue during the bone healing and remodeling of the osteoclasts. In this process, osteoclasts move to the microfracture areas of bone and secret hydrogen ions through the ruffled border, i.e., specialized membrane domains of the osteoclasts, into the microfracture and produce a localized acidic condition of about pH 4. This acidic condition resolves the mineral and organic matrix to help new bone formation^41–43^. Accordingly, we used an AcOH buffer (pH 4.2) to mimic the osteoclastic resorption of bone during the healing process based on the previous studies^44,45^.

The results showed that M-CNFs were not degradable in PBS (pH 7.4) for 60 days since there was no significant weight change. On the other hand, the data indicated that the fabricated M-CNFs were degradable and lost about 50% of their initial weight into the AcOH buffer (pH 4.2). Previous studies have revealed that, HA crystals are degradable in acidic conditions^46,47^. According to the obtained results and the reported data, the observed weigh loss can be related to the HA degradation, rather than CNFs degradation.

### Cell culture studies

#### Cell toxicity

**Figure 6:**
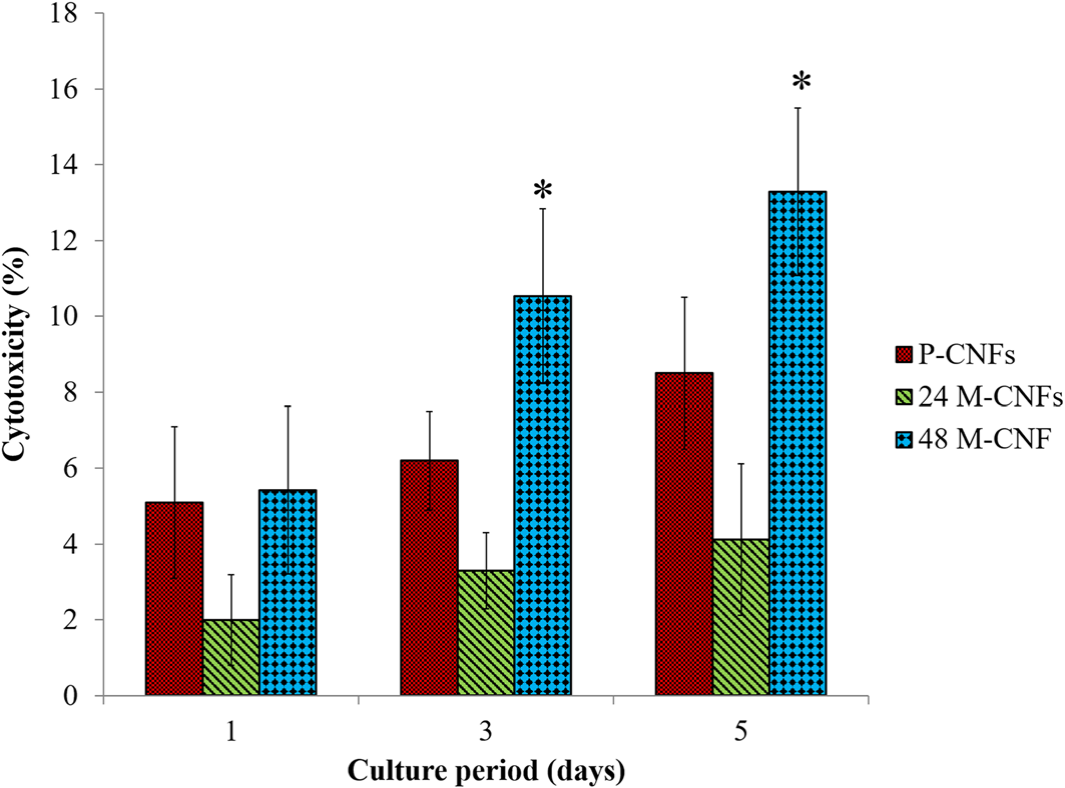
Cytotoxic effects of P-CNFs, 24M-CNFs, and 48M-CNFs on MG-63 cells, measured by lactate dehydrogenase (LDH) assay. MG-63 cells seeded on CNFs with a density of 5,000 cells per well in a 96-well plate and incubated for 1, 3, and 5 days. LDH activity was measured after cell lysis. Data represented as mean ± SD, n = 5. The results are given as relative values to the negative control (tissue culture plate, TCP) in percent, whereas negative control is set to be 0% cytotoxic. Asterisk indicates significant difference compared to the control (TCP).

The toxicity of the fabricated CNFs against MG-63 cells was evaluated using the LDH toxicity assay (Fig. 6). The results of the toxicity showed that, although the induced toxicity of P-CNFs was statistically significant and higher than the control group (TCP) (p < 0.005) on days 3 and 5, it was lower than 10%. Moreover, 24M-CNFs did not show any significant cytotoxicity (< 5%) even after 5 day cell seeding. These findings indicated that P-CNFs, and specifically the 24M-CNFs, are cytocompatible against MG-63 cells. Our previous study also showed that the toxicity of the pure PAN-based CNFs was in the acceptable range (below 10%) against the human endometrial stem cells (hEnSCs)^20^. Interestingly, 48-M-CNFs induced significant toxicity (p < 0.005) after 1, 3, and 5 days of cell culture. The 48M-CNFs induced the highest toxicity (13.53 ± 2.21%) after 5 days of cell culture.

#### Cell proliferation findings

**Figure 7:**
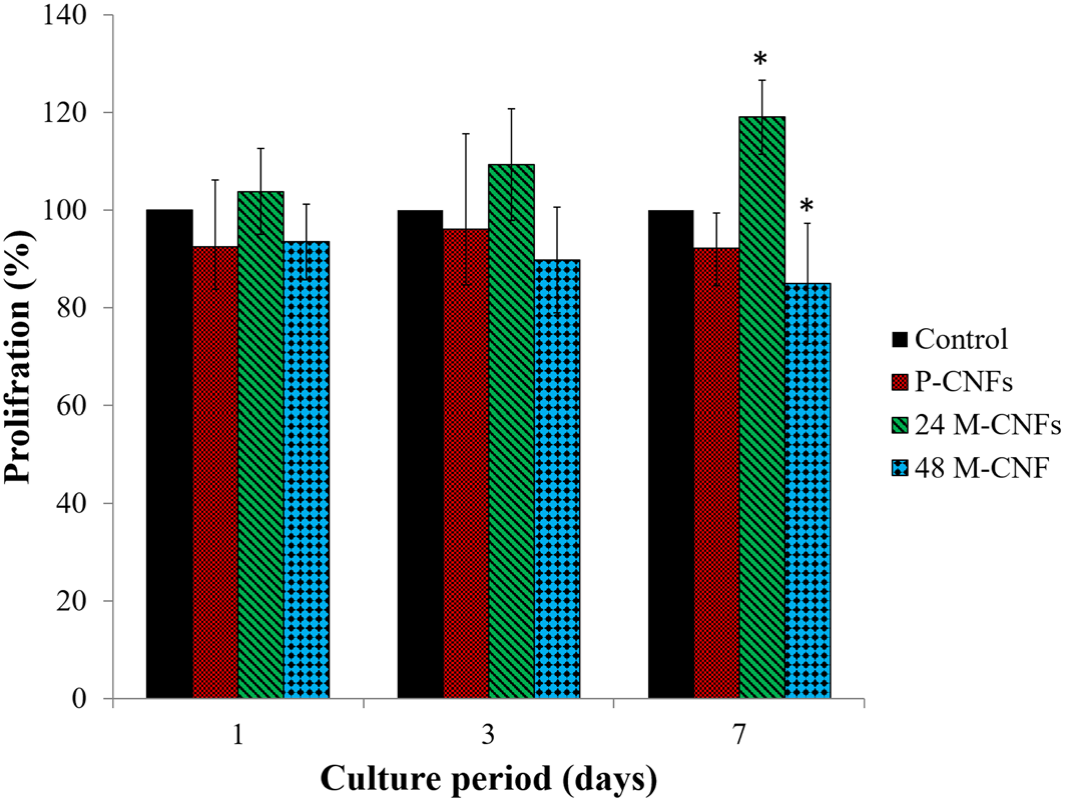
The proliferation of MG-63 cells on P-CNFs, 24M-CNFs, and 48M-CNFs measured by total lactate dehydrogenase (LDH) activity assay. The MG-63 cells seeded on CNFs with a density of 5,000 cells per well in a 96-well plate and incubated for 1, 3, and 7 days. LDH activity was measured after cell lysis. Data represented as mean ± SD, n = 5. The results are given relative values to the control (tissue culture plate, TCP) in percent, whereas the proliferation of cells on control is set to 100%. The asterisk indicates a significant difference compared to control (TCP).

The proliferation of MG-63 cells seeded on the fabricated CNFs/HA nanocomposite was further evaluated by the total LDH activity assay (Fig. 7). The proliferation assay results confirmed the toxicity assays findings, i.e., 24M-CNFs enhanced cell proliferation, while 48M-CNFs inhibited the growth of the cells (p < 0.05). Although, the effect of HA on cell viability is controversial, the positive effects of HA on the biocompatibility of various materials have been widely approved.

Various studies have reported the converse effects of HA on cell proliferation and viability. Chen and colleagues reported that HA induced cell toxicity and apoptosis in SGC-7901 cells in a dose-dependent manner^48^. They observed downregulation of Bcl-2, an antiapoptotic protein, overexpression of Bax pro-apoptotic protein, and leakage of cytochrome *c* from mitochondria into the cytoplasm. However, other researchers reported a dose-dependent cell proliferation inhibition rate (CPIR), reactive oxygen species (ROS) generation, p53 expression, apoptosis, and DNA fragmentation in MCF-7 cells treated with different doses of HA^49^. They suggested that HA at high dose induces oxidative stress in cells by activating the p53 gene and the related downstream genes such as PUMA, BAX, p21, GADD45, PCNA, and Bcl2 which are involved in the apoptotic pathways. These findings, along with our results, indicate that, although HA crystals are beneficial for cell proliferation, at the higher dose, they may induce some adverse effects.

#### Cell attachment and morphology

**Figure 8:**
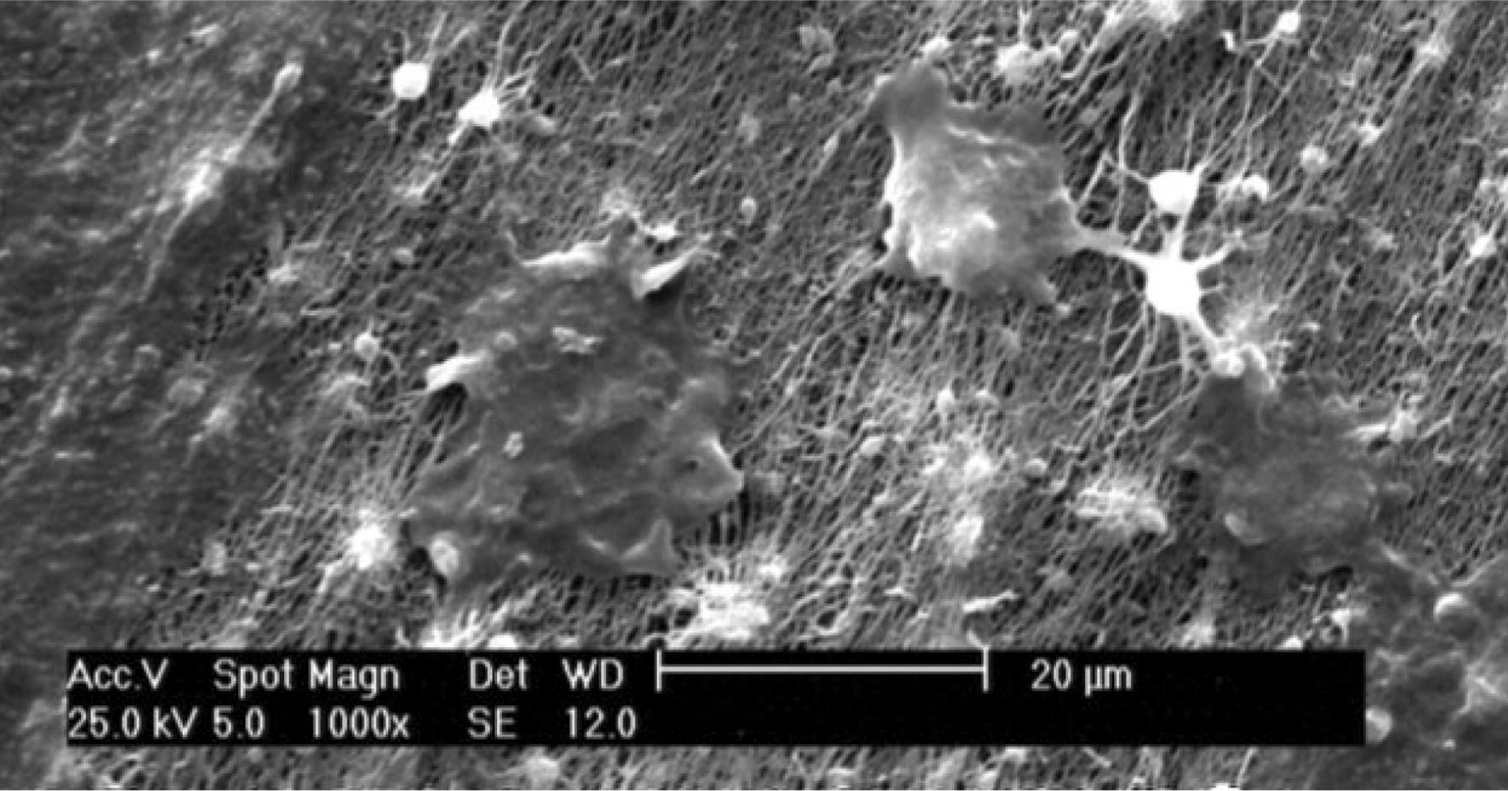
SEM micrograph of MG-63 cells on CNFs.

The morphology of MG-63 cells grown on M-CNFs was studied using SEM imaging (Fig. 8). MG-63 cells were well adhered to and spread on the CNFs, revealing the biocompatibility of CNFs surface for cell attachment. This observation is consistent with the results of the toxicity and proliferation assays.

### In vivo bone defect healing by CNFs/HA nanocomposite

#### CT images

**Figure 9:**
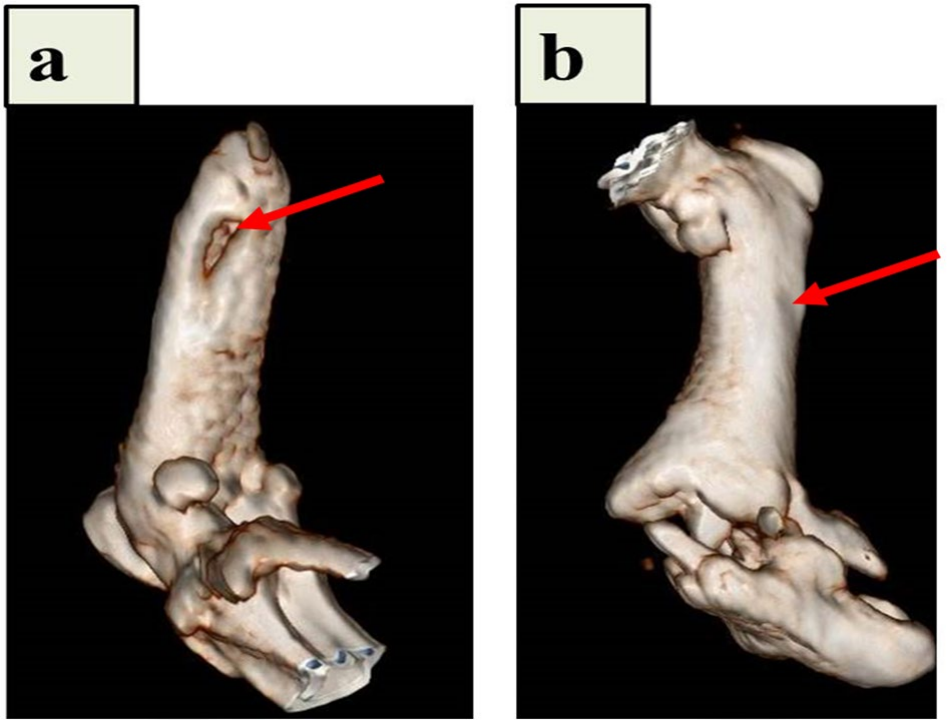
The CT images of in vivo repair of the defective femur by CNFs/HA nanocomposite implants. Diagnostic 3D imaging (CT scan) of the femur bone defects after 8 weeks of injury. The arrow shows the unrepaired defective site in the control group (**a**) and the bone defect repaired by the growth of normal tissue caused by the implanted CNFs/HA nanocomposite (**b**).

The induced bone tissue formation by CNFs/HA nanocomposite at the femur's defective site was studied using the 3D images obtained by CT scan 8 weeks after implantation (Fig. 9). The images showed that the defect was completely filled at 8 weeks post-implantation in the presence of CNFs/HA, whereas there was no healing identified in the non-treated control group, and thus, the defect had remained nearly the same.

#### Histological observation

**Figure 10:**
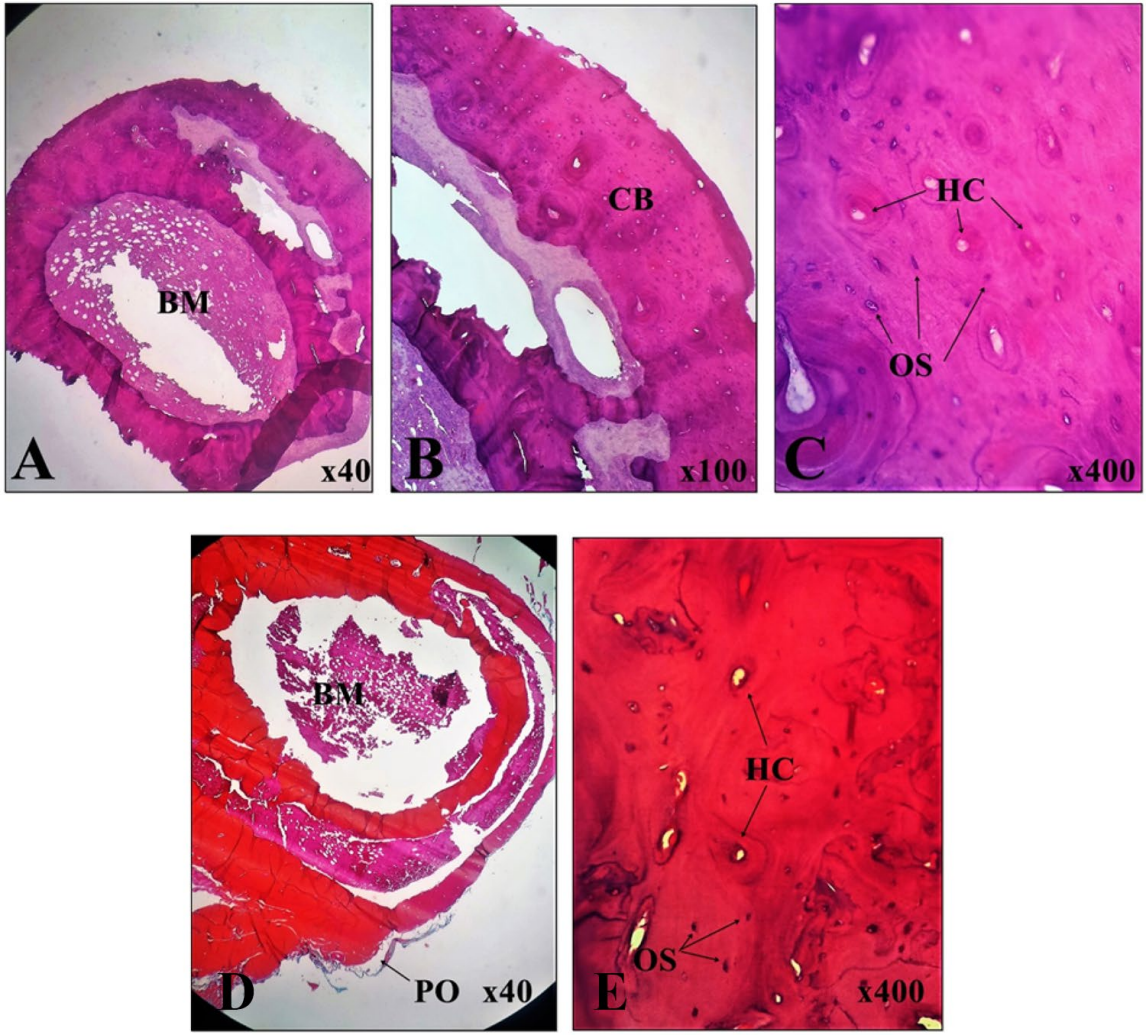
Histopathological section of the positive control group of normal femoral bone without any defect. *BM* bone marrow, *HC* Haversian canal, *OS* osteocyte, *CB* compact bone, *PO* periosteal layer, (**A**–**C**) H&E staining, (**D**,**E**) MT staining.

**Figure 11:**
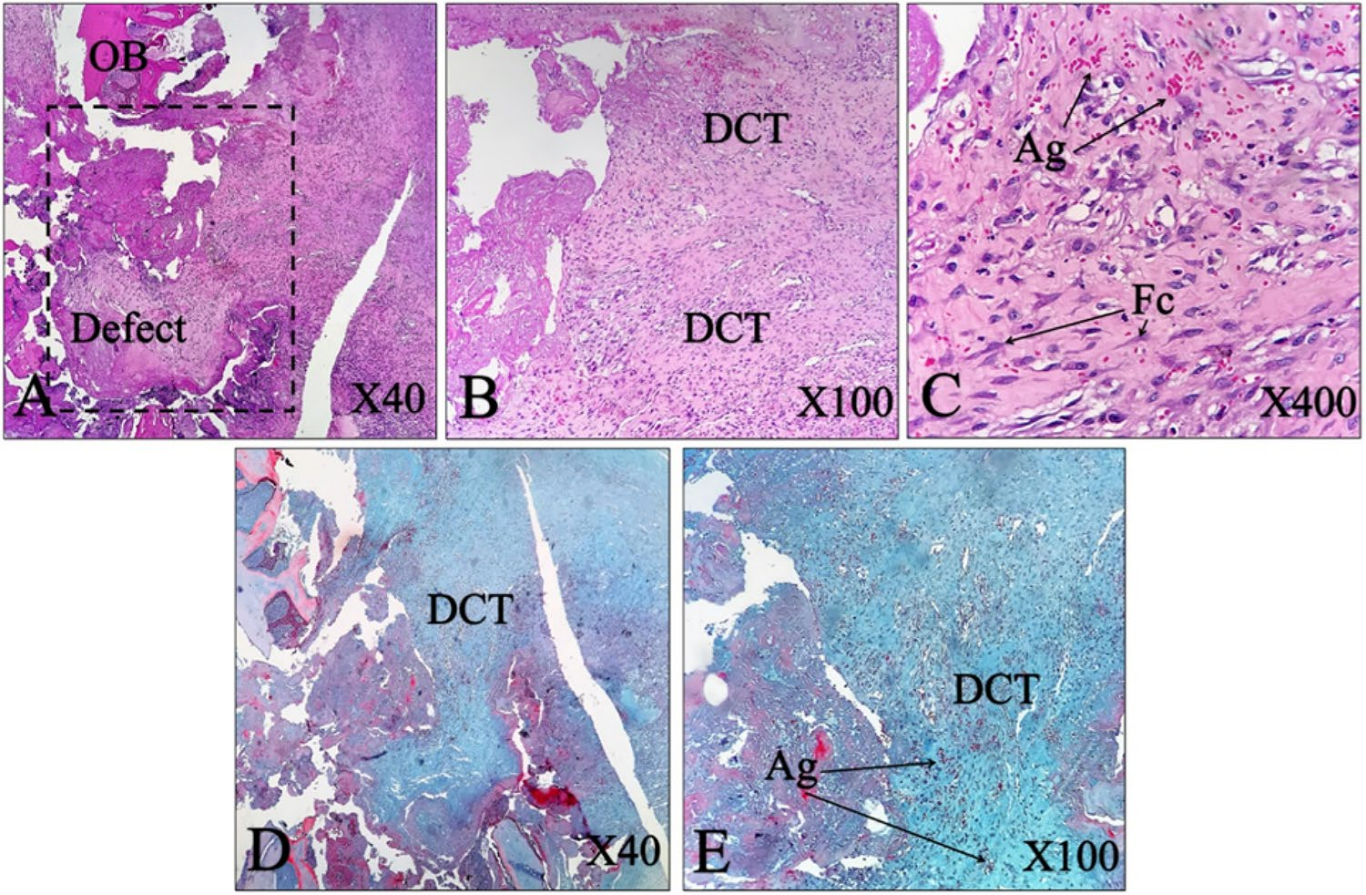
Histopathological section of the normal and defective bone. The negative control group shows the defect without any treatment. *OB* old bone, *DCT* dense connective tissue, *Ag* angiogenesis, *Fc* fibrocyte. (**A**–**C**) H&E stain, (**D**,**E**) MT stain.

The histopathological evaluation of the positive control group showed healthy bone tissue without any defect (Fig. 10). The defective site was filled with dense connective tissue (DCT) in the negative control group and no new bone formation (NBF) identified in this group, 2 months post-surgery (MPS) (Fig. 11). The result implies that the animal body was not capable of healing the untreated created defect after 2 months.

The histopathological assessment of the treated group in which the defect filled with CNFs, showed a small remnant of the CNFs in the defect area; however, the defect site was filled with hyaline cartilage and that the formation of new bone tissue was evident. The new bone formation was dominant in the CNFs treated group. Moreover, numerous macrophages, lymphocytes and giant cells appeared at the defect site. MT stating showed the marginal area of hyaline cartilage and newly formed woven bone, where the woven bone had been occupied with high numbers of different cells, including osteocytes (Fig. 11).

**Figure 12:**
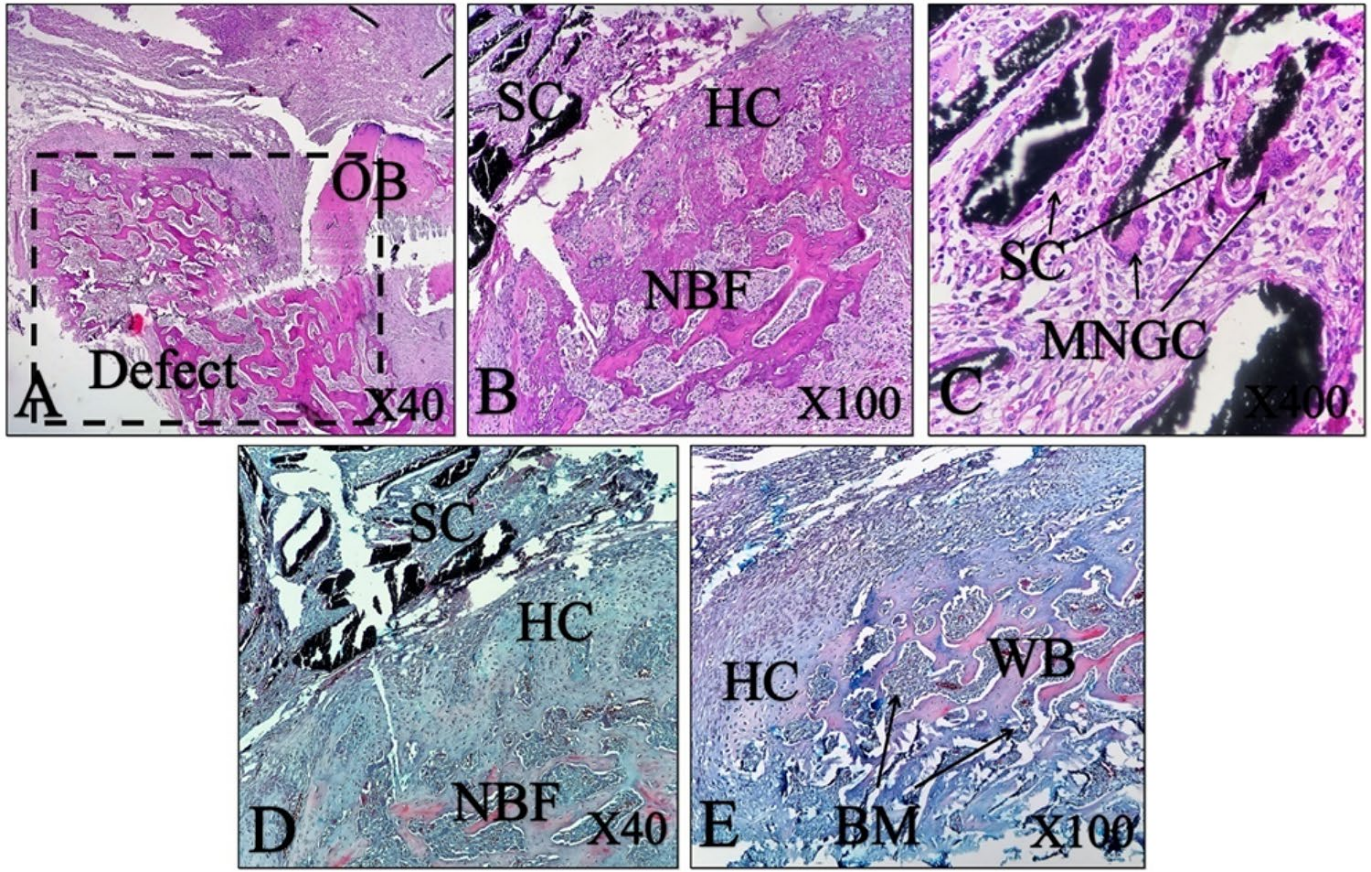
Histopathological section of bone tissue of a CNFs treated group. *SC* scaffold remnant, *OB* old bone, *HC* hyaline cartilage, *BM* bone marrow, *NBF* new bone formation, *MNGC* multinucliated giant cell, *WB* woven bone. (**A**–**C**) H&E stain, (**D**,**E**) MT stain.

The fabricated CNFs were biocompatible and showed osteoconductive characteristics, which induced cell growth in bone and facilitated the attachment of segregated cells (Fig. 12). Moreover, CNFs did not disturb the healing process and in comparison with the negative control group, they elicited bone formation and caused new bone ingrowth into the defect sites (Fig. 12).

The histomorphometric evaluation showed that CNFs induced more cartilaginous and osseous tissue formation in the defect area than the negative control group. On the contrary, the density of fibrous connective tissues and immature granulation tissue and cells (i.e., number of fibrocytes and fibroblasts, the density of collagen fibers and the newly regenerated blood vessels) was significantly higher in the negative control group compared with the CNFs treated group (p < 0.01). Analysis of the CT scan images in combination with histopathological and histomorphometric findings revealed that CNFs were able to induce bone formation in the defect site (Table 2).

**Table 2:** Histomorphometric findings of bone tissue regeneration in the defect area.

Valuable	Negative control	M-CNF
Fibroblast + fibrocyte	235.2 ± 18.6	60.4 ± 5.5
Chondroblast + chondrocyte	3.5 ± 1.2	34.1 ± 4.7
Osteoblast + osteocyte	14.1 ± 3.5	85.8 ± 9.0
Osteoclast	0	8.0 ± 1.7
Osteon	2.1 ± 0.54	19.7 ± 2.6
NBF (%)	0	61.3 ± 4.2

## Conclusions

Tissue engineering is considered an effective alternative means of treatment over the conventional therapy of bone defects. ECM of bone tissue composed of collagen nanofibers reinforced and composited with HA crystals. Hence, considerable attention has been drawn to the fabrication of a composite of nanofibers and bioceramics possessing similar porosity and surface characteristics. Here, we aimed to fabricate CNFs/HA nanocomposite with the maximum resemblance to that of bone ultrastructure and used to heal the intentionally mechanically made defects in the rat femur. The results showed that the HA crystals were formed homogeneously around the treated CNFs through the applied biomimetic approach. Based on the results of XRD and using the Sherrer equation, the crystal size of HA was calculated at around 35.2 nm, which is comparable with the crystal size of HA in the natural bone. FTIR spectroscopy also confirmed carbonate-containing HA formation on the CNFs. EDAX elemental analysis revealed that the mineral phase formed onto the CNFs is mainly composed of O, P, and Ca, the main elements of HA crystal. It was observed that treating CNFs with concentrated NaOH reduced the mechanical strength of CNF from 6.32 ± 0.10 to 6.01 ± 0.34 MPa. The mineralization process enhanced mechanical strength, which was not statistically significant (p < 0.1). Furthermore, the mineralization process turned the hydrophobic CNFs, 133.5° ± 0.6°, to the superhydrophilic, 0°, after 72 h mineralization process. The toxicity and proliferation assessments showed that 24M-CNFs was biocompatible with negligible toxicity, while 48M-CNFs induced the highest toxicity, 13.53 ± 2.21%. The in vivo studies depicted that M-CNFs induced 61.3 ± 4.2% new bone formation, which was statistically significant (p < 0.005) compared with the negative control group. In conclusion, our study confirmed the efficacy of CNFs/HA nanocomposite as the osteoconductive and electroactive scaffolds for bone tissue engineering applications and can be considered as the bone healing biomaterials.

## Materials and methods

### Materials

Poly(acrylonitrile) (PAN, MW: 80,000 gmol^−1^, Polyacryl Company, Esfahan, Iran), *N*,*N*-dimethylformamide (DMF, Merck, Darmstadt, Germany), MG-63 cell line (National Cell Bank of Iran (NCBI), Pasteur Institute, Tehran, Iran), Dulbecco’s modified Eagle’s medium: nutrient mixture F-12 (DMEM/F12, Gibco, USA), 3-(4,5-dimethylthiazol-2-yl)-2,5-diphenyltetrazolium bromide (MTT, Roche, Germany), fetal bovine serum (FBS, Gibco, USA), and Lactate dehydrogenase (LDH) assay kit (LDH Cytotoxicity Detection KitPLUS, Roche, Germany) were used in the current study.

### CNFs fabrication

Based on our previous study^32^, CNFs were fabricated from the heat treatment of electrospun PAN nanofibers. Briefly, PAN nanofibers were fabricated through electrospinning a solution of PAN/DMF (10% w/v) using a commercial electrospinning apparatus (Electronics, FNM, Tehran, Iran). During the electrospinning, the applied voltage, the polymer solution feeding rate, and nozzle to collector distance were set as 20 kV, 1 ml min^−1^, and 10 cm, respectively. The fabricated PAN nanofibers were converted to the CNFs via two steps of heat treatment, stabilization and carbonization. The stabilization was conducted at 290 °C for 4 h with a heating rate of 1.5 °C min^−1^ under air atmosphere, while carbonization was done in a high-purity nitrogen atmosphere at 1,000 °C for 1 h with the heating rate of 4 °C min^−1^ using a tube furnace (Azar, TF5/25-1720, Iran) ^50^.

#### Carbon nanofibers mineralization

The biomimetic mineralization process was used to grow mineral phase at the surface of the fabricated CNFs^27^. First, the surface of CNFs was activated via concentrated NaOH solution (5 mol l^−1^) for 24 h at 45 °C to induce formation of carbonyl functional groups on the surface of CNFs. The formed carbonyl functional group served as the mineral phase nucleation site (Fig. 1). The resulted surface activated CNFs were soaked in SBF while shaking for 12, 24, 48, and 72 h at 37 °C. The SBF solution was refreshed on a daily basis and the resulted modified CNFs (M-CNFs) removed from solution at the end of mentioned times, thoroughly rinsed with deionized water, dried at 80 °C for 24 h, and stored in a cool and dry place for further use and evaluation.

### Characterization

#### Morphological observation

The morphology of the fabricated CNFs was observed by scanning electron microscopy (SEM, Philips XL-30, Germany). CNFs were sputter coated with a thin layer of gold using a sputter coater (SCD 004, Balzers, Germany) and observed at 20 kV accelerating voltage.

#### Mineral phase evaluation

The formed mineral phase on the surface of CNFs was further evaluated using SEM-EDAX elemental analysis (XL 30, Philips), X-ray diffraction (XRD) of CuKα radiation (λ = 1.54056 Å, 40 kV, 30 mA, the step size of 0.08°s^−1^ over the 2θ at 10°–90°) using a Philips Xpert instrument, and finally by Attenuated Total Reflectance–Fourier Transform Infrared (ATR–FTIR) spectroscopy.

#### Mechanical properties evaluation

Tensile strength measurement was conducted based on ISO 5270:1999 standard test methods to assess the mechanical properties of the fabricated M-CNFs. The measurement was performed using a uniaxial tensile testing device (Santam, Karaj, Iran) at a strain rate of 10 mm/min.

#### Wettability assessment

Water contact was measured as the function of wettability of the fabricated M-CNFs via a static contact angle measuring device (KRUSS, Hamburg, Germany). The angle between the poured water droplet and the surface of CNFs was measured at three different points, averaged, and reported.

#### Biodegradation measurement

The biodegradation study was conducted based the weight loss measurement of the CNFs during 60 days in two different acidic conditions, (PBS, pH 7.4) and acetate sodium/acetic acid buffer (pH 4.2). The degradation process further assessed by SEM imaging during the culture period.

### Cell culture studies

#### Cell toxicity assay

The toxicity measurement was done by the LDH cytotoxicity assay kit, which evaluated the intactness of the cell membrane through the measuring the LDH enzyme leaked from the damaged cell membrane. The CNFs were cut circularly, put on the bottom of the 96-well plates, sterilized with ethanol (70.0%) for 2 h, washed thoroughly with sterile PBS (pH 7.4), and exposed to the UV light, overnight. A number of 5,000 cells in 100 μl of DMEM/F12 medium supplemented with FBS (1.0% v/v) and antibiotic (1.0%) were seeded on the triplicates of carbon mats and tissue culture plastics (TCP) as a control. The plates were incubated for 1, 3 and 5 days at 37 °C in a humidified atmosphere in the presence of 5.0% CO2 and the toxicity was measured according to the procedure described by the manufacturer (Eq. 1)

1 $$Cytotoxicity\; (\% ) = \frac{exp.\; value - low \;control }{{high\; control - low \;control}} \times 100$$

LDH activity released from the untreated cells and the maximum releasable LDH activity in the cells were considered as Low control and high control, respectively. Here, high control value, was obtained by culturing cells on TCP and lysing them by the reagent at the end of each time point. The percent of toxicity was relative to the TCP.

#### Cell proliferation measurement

The total LDH content of the cells was measured as a function of cell proliferation by the LDH assay after 1, 3 and 5 days cell seeding. At the end of each time point, the culture medium was removed and CNFs mat washed with PBS and lysed with the lysis solution (5 μl/100 μl culture medium). The total LDH release in each sample (test and control) was measured at 490 nm and the corresponding proliferation was calculated (Eq. 2):

2 $$Proliferation\; (\% ) = \frac{exp. \;value}{{control \;value}} \times 100$$

The proliferation of cells in the control groups was set to 100% and the percentage of proliferation in test groups was measured, accordingly (TCP).

#### Cell attachment  analysis

The morphology of cells cultured on the surface of CNFs was observed using SEM imaging. Briefly, after 24 h the seeded cells were fixed in paraformaldehyde (4% w/w) for 1 h at room temperature, dehydrated in upgrading ethanol, sputter coated with a thin layer of gold, and observed at an accelerating voltage of 26 kV.

### Animal studies

#### Bone defect creation

The animal studies were performed on ten adult male Wistar rats. Animal experiments were carried out in accordance with the guidelines of the Tehran University of Medical Sciences and approved by the ethical committee of the university. The animals were anesthetized by IP injection of xylazine:ketamine both from Alfasan, Woerden, the Netherlands, in a 2 µl and 40 µl per gram of body weight of rats, respectively. In order to form a bone defect, a 6 mm segmental damage was created in the rat femurs by the trephine (Meisinger) at a speed of 1,000 rpm under continuous irrigation with sterile saline and stabilized^33^. Briefly, a longitudinal incision was made over the left anterolateral femur and the entire femoral shaft was exposed using blunt dissection. The rats were then randomly divided into two groups of 5, the control (defect with no treatment) and CNFs treated group. Subsequently, 30 mg of the M-CNFs was embedded into the defect area and the periosteum was repositioned and closed with nylon suture (No. 6.0, SUPA medical devices, Tehran, Iran). The musculature and skin were closed with No. 3.0 polyglycolic acid and nylon sutures (SUPA medical devices, Tehran, Iran), respectively.

#### 3D Computed Tomography (CT) imaging

The bone formation was evaluated by CT-scan imaging 8 weeks post-implantation, using Inveon unit (Siemens Healthcare, Inc., PA, USA). The specimens were scanned at multiple longitudinal and transverse sections and the 3-D images of the newly formed bone tissues was created by the Inveon Research Workplace software (Siemens Healthcare USA, Inc., PA, USA).

#### Histological assessments

The animals were euthanized 2 months post-operation and their femoral bones were fixed in neutral buffered formalin (NBF, 10%; pH 7.26) for 48 h. The treated bones were decalcified by storage in nitric acid (5% v/v) for 10 days. The decalcified bones, then went through histological fixation and dehydration processes, and embedded in paraffin. Sections were cut (5 µm thick) and stained with haematoxylin and eosin (H&E) and Masson's trichrome (MT). The resulted histological slides were evaluated by a pathologist, using light microscopy (Olympus BX51; Olympus, Tokyo, Japan). The extent of the newly formed cartilage or bone as well as the size of the remaining implants in the total area of the section was assessed. Furthermore, the histo-morphometric analysis was carried out and the number and morphology of different cells including; fibroblasts, fibrocytes, chondroblasts, chondrocytes, osteoblasts, osteocytes, giant cells, macrophages, lymphocytes, neutrophils, as well as other constituents such as blood vessels, and newly formed cartilage and bone tissues were studied. The resulted data were analyzed using Image-Pro Plus V.6 (Media Cybernetics, Inc., Silver Spring, USA).

### Statistical analysis

All experiments were conducted in triplicate, except for the toxicity and proliferation assays which repeated five times for each sample and the results were reported as a mean ± standard deviation (SD). The statistical significance of the data was analyzed by Single factorial analysis of variance (ANOVA) using Statistical Package for Social Sciences software version 10.0 (SPSS 10.0). The significance level was set at p < 0.05.

### Ethical approval

The study was approved by the Ethics Committee in Tehran University of Medical Sciences. The methods in the study were in accordance with the guidelines of the Declaration of Helsinki. The animal study was conducted on 10 male adult Wistar rats after approval of the ethics committee of Tehran University of Medical Sciences. All applicable international, national and institutional guidelines for the care and use of animals were followed.

## Data Availability

The datasets generated during and/or analysed during the current study are available from the corresponding author on reasonable request.
